# *Onchocerca volvulus* and epilepsy: A comprehensive review using the Bradford Hill criteria for causation

**DOI:** 10.1371/journal.pntd.0008965

**Published:** 2021-01-07

**Authors:** Robert Colebunders, Alfred K. Njamnshi, Sonia Menon, Charles R. Newton, An Hotterbeekx, Pierre-Marie Preux, Adrian Hopkins, Michel Vaillant, Joseph Nelson Siewe Fodjo

**Affiliations:** 1 Global Health Institute, University of Antwerp, Antwerp, Belgium; 2 Neurology Department, Yaoundé Central Hospital, Yaoundé, Cameroon; 3 Faculty of Medicine and Biomedical Sciences, University of Yaoundé I, Yaoundé Cameroon; 4 Brain Research Africa Initiative (BRAIN), Yaoundé, Cameroon; 5 Department of Psychiatry, University of Oxford, Oxford, United Kingdom; 6 Institute of Epidemiology and Tropical Neurology, INSERM UMR1094, University of Limoges, Limoges, France; 7 Neglected and Disabling Diseases of Poverty Consultant, Kent, United Kingdom; 8 Competence Center in Methodology and Statistics, Luxembourg Institute of Health, Strassen, Luxembourg; National Institutes of Allergy and Infectious Diseases, NIH, UNITED STATES

## Abstract

**Background:**

The possibility that onchocerciasis may cause epilepsy has been suggested for a long time, but thus far, an etiological link has not been universally accepted. The objective of this review is to critically appraise the relationship between *Onchocerca volvulus* and epilepsy and subsequently apply the Bradford Hill criteria to further evaluate the likelihood of a causal association.

**Methods:**

PubMed and gray literature published until September 15, 2020, were searched and findings from original research were synthesized. Adherence to the 9 Bradford Hill criteria in the context of onchocerciasis and epilepsy was determined to assess whether the criteria are met to strengthen the evidence base for a causal link between infection with *O*. *volvulus* and epilepsy, including the nodding syndrome.

**Results:**

Onchocerciasis as a risk factor for epilepsy meets the following Bradford Hill criteria for causality: strength of the association, consistency, temporality, and biological gradient. There is weaker evidence supporting causality based on the specificity, plausibility, coherence, and analogy criteria. There is little experimental evidence. Considering the Bradford Hill criteria, available data suggest that under certain conditions (high microfilarial load, timing of infection, and perhaps genetic predisposition), onchocerciasis is likely to cause epilepsy including nodding and Nakalanga syndromes.

**Conclusion:**

Applying the Bradford Hill criteria suggests consistent epidemiological evidence that *O*. *volvulus* infection is a trigger of epilepsy. However, the pathophysiological mechanisms responsible for seizure induction still need to be elucidated.

## Author summary

There is growing epidemiological evidence that an infection with *Onchocerca volvulus* (the parasite causing onchocerciasis or river blindness) can trigger epilepsy, including nodding and Nakalanga syndromes. We studied the association between onchocerciasis and epilepsy using previously proposed criteria for causality and found strong arguments in favor of a causal relationship. The risk for children to develop epilepsy seems to be determined by the *O*. *volvulus* microfilarial load. However, the mechanism by which the *O*. *volvulus* parasite is able to trigger seizures remains to be elucidated. This form of epilepsy, called onchocerciasis-associated epilepsy (OAE), is an important public health problem in onchocerciasis-endemic regions with nonexistent or suboptimal onchocerciasis elimination programs. OAE is often characterized by sudden seizure onset in previously healthy children, between 3 to 18 years, without any other obvious cause of epilepsy. On the basis of the findings presented in this paper, it appears that onchocerciasis control may be able to prevent OAE. This further highlights the importance to eliminate onchocerciasis, particularly in areas with a high burden of epilepsy.

## Introduction

The possibility that onchocerciasis may cause epilepsy has been suggested since the pioneering work of Casis-Sacre in 1938 [1–5]; however, an etiological link is still pending universal acceptance. Hitherto, *Onchocerca volvulus* microfilariae, the small first-stage larval progeny produced by the adult female worms which are responsible for the disease manifestations of onchocerciasis (river blindness), have not been found in the brain parenchyma. However, microfilariae have been detected in cerebrospinal fluid in the past particularly after treatment with diethylcarbamazine [6,7]. *O*. *volvulus* is transmitted by tropical species of Simulium blackflies, which breed in fast-flowing waters, delimiting the distribution of onchocerciasis to areas in proximity to rivers in sub-Saharan Africa (SSA), some countries in Latin America, and Yemen. Epidemiological studies also suggested a potential link between nodding syndrome (NS), an epileptic encephalopathy, and onchocerciasis [8,9]. The pathogenesis of NS was recently discussed in an excellent review paper [10]. However, no systematic review that considers all the available original research data concerning the association between epilepsy including NS and onchocerciasis has so far been performed.

In 1965, Bradford Hill proposed 9 criteria for evaluating traditional epidemiologic data to “pass from an observed association to a verdict of causation,” namely: strength of association, consistency, specificity, temporality, biological gradient, plausibility, coherence, experimental evidence, and analogy [11]. Building on the 9 criteria, other researchers have added excluding confounding factors and bias [12]. Since then, the Bradford Hill criteria have been frequently used in epidemiological studies to demonstrate causal inference, for example, Zika virus and microcephaly [13], sugar-sweetened beverages and coronary heart disease [14], and chrysotile asbestos and mesothelioma [15].

In this paper, we conduct a comprehensive review of published and unpublished literature and use Bradford Hill criteria to gauge the causality of association between infection with *O*. *volvulus* and epilepsy, including NS. We also highlight the criteria for which evidence is weaker or absent and identify research needs.

## Material and methods

We searched for articles indexed in PubMed until September 15, 2020. Search terms used included “epilepsy,” “nodding syndrome/seizures,” “Nakalanga syndrome/features,” and “onchocerciasis” (see S1 Table). A manual search to identify relevant published articles as well as unpublished gray literature such as postgraduate research theses/dissertations was also performed. All original research reporting relevant quantitative findings were included in our review, irrespective of the study design. Data were extracted from eligible studies and organized in electronic spreadsheets.

### Causal association assessment

The possibility of a causal relationship between onchocerciasis and epilepsy (including nodding and Nakalanga syndromes [16]) was investigated by applying each of the 9 Bradford Hill criteria adapted to the case of onchocerciasis and epilepsy. We also discussed potential confounding factors and bias in the retrieved documentation (Table 1).

**Table 1:** Bradford Hill criteria for assessing causation [11] and their adaptation to the case of onchocerciasis and epilepsy.

Criteria	Bradford Hill criteria [11]	Adaptation to *O*. *volvulus* infection and epilepsy, including NS and Nakalanga
**1. Strength of association**	The larger the association, the more likely that it is causal	The strength of the association between onchocerciasis and epilepsy/NS in studies carefully conducted to adjust for relevant confounders such as ivermectin use. An odds ratio >3.0 was considered as a strong association
**2. Consistency**	Consistent findings observed by different persons in different places with different samples strengthens the likelihood of an effect	The association between onchocerciasis and epilepsy/NS is replicated by different researchers in different countries/localities with different samples and where the net effect points toward the same direction
**3. Specificity**	Specificity is considered if an exposure is causing a very specific disease in a specific population at a specific site and with no other likely explanation. The more specific an association between an exposure and an effect, the bigger the probability of a causal relationship	Causation of epilepsy/NS can be considered if *O*. *volvulus* infection is causing the very specific disease manifestations in a specific population at a specific site with no other likely explanation, and if elimination of onchocerciasis eliminates incident cases of epilepsy/NS
**4. Temporality**	The effect has to occur after the cause	Epilepsy/NS occurs only following a documented infection with *O*. *volvulus*
**5. Biological gradient**	Greater exposure should generally lead to greater incidence of the effect	Greater prevalence (endemicity) and/or infection intensity of onchocerciasis should lead to greater incidence/severity of epilepsy/NS
**6. Plausibility**	A plausible biological mechanism between cause and effect is helpful	A plausible biological mechanism explaining how *O*. *volvulus* infection may cause epilepsy/NS
**7. Coherence**	Coherence between epidemiological and laboratory findings increases the likelihood of causality. Similar to biological plausibility, cause and effect should be in line with the knowledge available within the scientific community	Coherence between epidemiological and parasitological findings and the natural history of onchocerciasis and epilepsy/NS
**8. Experiment**	Experimental evidence from laboratory studies or randomized clinical trials	Experimental evidence (e.g., from animal models) showing that *O*. *volvulus* infection can lead to epilepsy/NS. Randomized clinical trials showing that treatment of onchocerciasis decreases the incidence of epilepsy/NS
**9. Analogy**	Causality is supported by analogy if there are similar associations or causal relationships in other areas of relevance; weakest form of evidence of causality	A similar pathophysiological mechanism is involved as in other onchocerciasis-associated conditions such as skin and eye disease

## Results

**Fig 1:**
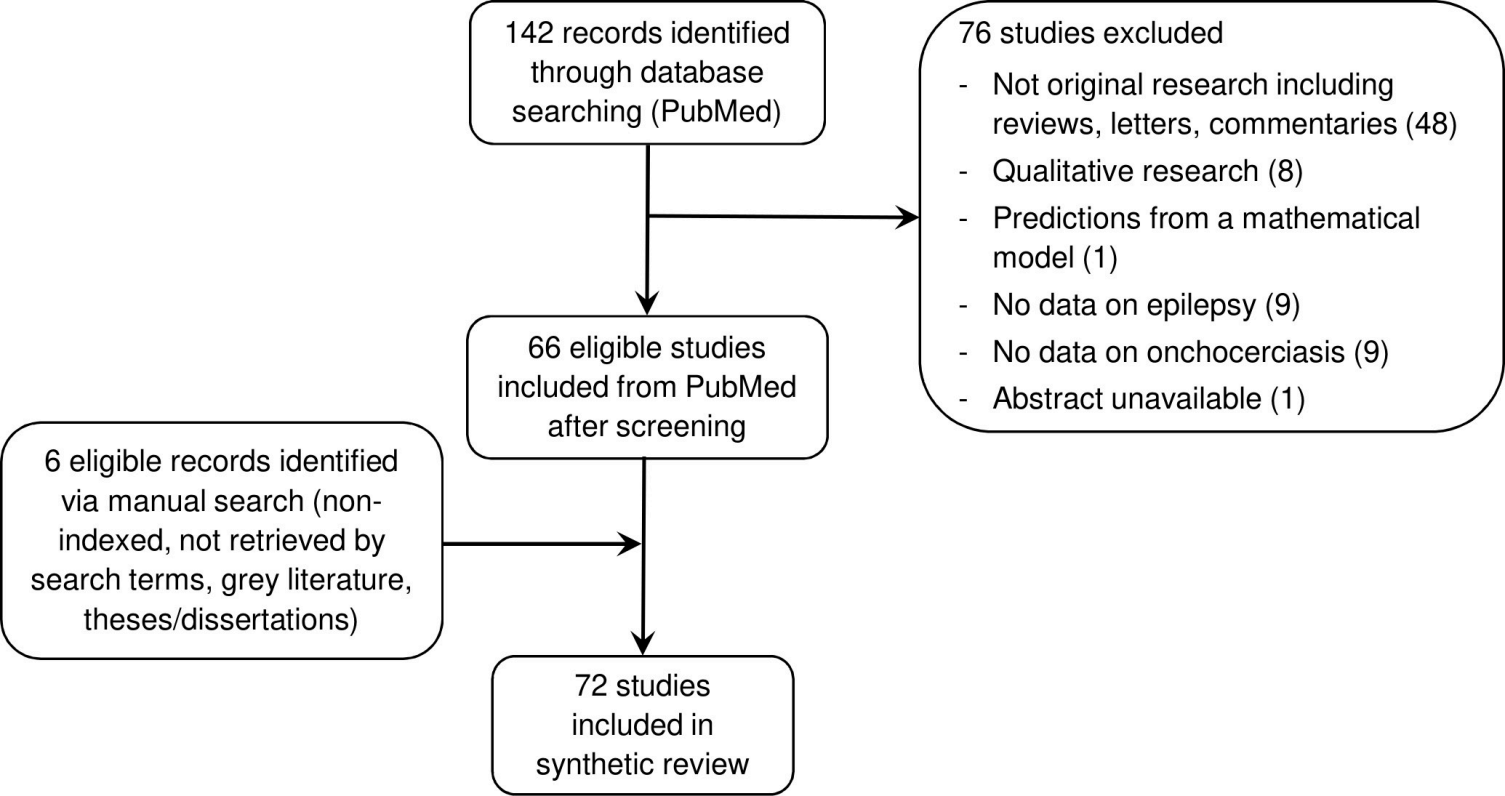
Selection of eligible studies.

We retrieved 142 studies from PubMed (Fig 1). After screening the abstracts, 66 studies were eligible for inclusion in our review. Six additional findings from manual searches were included as well (S2 Table).

### Strength of the association

The strength of association has been examined in case-control studies, 2 meta-analysis, and 2 cohort studies [17,18]. In case-control studies, *O*. *volvulus* antibodies [8,9,19], skin snip positivity [9,19], high skin microfilarial load [3,19], onchocerciasis-associated skin disease [19,20], and presence of nodules [21,22] were observed more frequently in persons with epilepsy (PWE) than in controls.

Druet-Cabanac and colleagues reported no statistical association between *O*. *volvulus* infection and epilepsy in their meta-analysis of 9 papers searched until 2002 (odds ratio (OR): 1.21; 95% confidence interval (CI 0.99 to 1.47; *p* = 0.06) [23]. Kaiser and colleagues reported an OR of 2.5 with a 95% CI of 1.6 to 3.9 for an association between epilepsy and onchocerciasis in their meta-analysis of 11 case-control studies published by 2012, but after controlling for age, sex, and place of residence, the OR became nonsignificant (1.3; 95% CI 0.9 to 1.8) [24]. Taking into account these meta-analyses [23,24], one may conclude there is no association between onchocerciasis and epilepsy. However, 2 of the 9 studies included in the meta-analysis by Druet-Cabanac and colleagues are problematic with regard to the investigation of the association between *O*. *volvulus* infection and epilepsy. One study was performed in a village that was already hypo-endemic following more than 14 years of vector control activities by the Onchocerciasis Control Programme in West Africa (OCP) [25]. The other study by Ovuga and colleagues considered participants with retarded growth as non-epileptic controls [2]. However, persons with retarded growth are not appropriate controls, as growth retardation itself might be onchocerciasis-related [26]. If these 2 studies were to be excluded from the meta-analysis, a relative risk of 1.30 (95% CI 1.04 to 1.62; *p*  =  0.02) would be obtained [27]. The OR reported in the meta-analysis by Kaiser and colleagues also needs to be interpreted with caution as case-control studies in hypo-endemic areas should be excluded from analysis because in such areas, the cause of epilepsy will be unrelated to onchocerciasis in the majority of PWE. In hyper-endemic areas that have not been exposed to ivermectin, it may be difficult to demonstrate an association between skin snip positivity and epilepsy because in such communities, almost everyone is infected. However, in such hyper-endemic settings, the intensity of *O*. *volvulus* infection (assessed via the microfilarial load) is often significantly higher among PWE compared to controls as demonstrated in the study by Boussinesq and colleagues [3]. Therefore, in the absence of past ivermectin treatment, a better parameter to compare PWE and controls would either be the microfilariae density or the number of nodules, both of which are proxies for the intensity of the onchocercal infection. A matched case-control study in an ivermectin-naïve population in an onchocerciasis-endemic region in Cameroon [3], and another in the Logo health zone in Ituri, Democratic Republic of Congo (DRC) [19], showed a higher microfilariae density among PWE compared to controls: 288 versus 141 microfilariae/skin snip, *P* < 0.0001 in the Mbam valley (Cameroon) and 31.8 versus 2.7 microfilariae/skin snip, *P* < 0.001 in Draju, Logo health zone in the DRC.

In case of many years of community-directed treatment with ivermectin (CDTI) in a hyper-endemic area, the interpretation of results may be difficult because past ivermectin intake between cases and controls may have been different. Considering case-control studies conducted in meso- and hyper-endemic villages without previous exposure to ivermectin, a significant association between onchocerciasis and epilepsy/NS with an OR >3 was reported in almost all studies (Tables 2 and 3). The strongest association was observed in a study that included only NS cases (Table 2) [9]. This was to be expected because NS is a specific clinical entity reported to occur only in onchocerciasis foci, whereas other forms of epilepsy occur everywhere and can result from etiologies unrelated to *O*. *volvulus*.

**Table 2:** Case-control studies investigating the association between onchocerciasis and nodding syndrome.

Author, year of study, study site	Pre-control onchocerciasis endemicity level	Number of years of onchocerciasis control	Onchocerciasis endemicity level at the time of the study	OR^#^ (95% CI) for presence of mf in cases vs controls
Foltz et al, 2009, Kitgum, Uganda [8]	Meso	0*	Meso	2.11 (0.86–5.2)
Tumwine et al, 2001, Amadi, South Sudan [9]	Hyper	Interrupted CDTI	Hyper	29.0 (3.5–237.7)
Tumwine et al, 2001, Lui, South Sudan [9]	Hyper	Interrupted CDTI	Hyper	9.3 (2.7–32.6)
Tumwine et al, 2002, Lui, South Sudan [9]	Hyper	Interrupted CDTI	Hyper	15.4 (1.6–148.8)
CDC, 2011, Maridi, South Sudan [28]	Hyper	Interrupted CDTI	Hyper	9.3 (1.9–52.3)
CDC, 2011, Witto, South Sudan [28]	Hyper	NA	Meso/hyper	1.0 (0.2–6.2)

^#^OR, unadjusted odds ratio.

Meso, meso-endemic (35% < mf prevalence < 60%); Hyper, hyper-endemic (mf prevalence >60%).

*33% of cases and 25% of controls had been treated with ivermectin.

CDC, Centers for Disease Control and Prevention; CDTI, community-directed treatment with ivermectin; mf, microfilariae; NA, not available.

**Table 3:** Case-control studies in meso- and hyper-endemic areas on the onchocerciasis-epilepsy relationship (ranked according to increasing duration of onchocerciasis control.

Author, year of study, study site	Pre-control onchocerciasis endemicity level	Number of years of onchocerciasis control	Onchocerciasis endemicity level at the time of the study	OR^#^ (95% CI) for presence of mf in cases vs controls
Boussinesq et al, 1991, Mbam, Cameroon [3]	Hyper (Pmf >69%)	0	Hyper (Pmf >69%)	4.18 (0.46–38.3)*
Newell et al, 1994, Bururi Province, Buyengero & Burambi, Burundi [29]	Meso-hyper	0	Meso/hyper	2.49 (1.38–4.5)
Mandro et al, 2015, Draju, Logo health zone, Ituri Province, DRC [19]	Meso	0	Meso	3.58 (1.68–7.63)
Kohler, 2000, Sanaga maritime, Cameroon [30]	Hyper	1	Hyper (Pmf >80%)	3.76 (1.31–10.74)
Kipp et al, 1993, Kabarole, Uganda [31]	1 hyper, 1 hypo	2	1 hyper, 1 hypo	7.31 (3.19–16.73)
Burfeind et al, 2014, Kasangulu, Bas-Congo Province, DRC [32]	Meso-hyper?	2	Meso-hyper?	4.51 (1.75–11.65)
Kaiser et al, 1994, Kabarole, Uganda [33]	7 hyper, 6 meso/hypo	3	7 hyper, 6 meso/hypo	1.67 (0.61–4.57)
Mandro et al, 2015, Rassia, Rethy health zone, Ituri Province, DRC [19]	Meso	3	Meso	2.13 (0.83–5.4)
Druet-Cabanac et al, 1996, Ouham and Ouham-Pende, Central African Republic [34]	Meso-hyper	5	Meso/hyper	1.17 (0.82–1.68)
König et al, 2005, Mahenge, Tanzania [35]	Meso-hyper	8	Meso	4.36 (2.63–7.24)
Gbenou, 1995, Agbogbome, Dassa-Zoumé, Benin [36]	Meso-hyper	8 (1 CDTI, 7 VC)	Meso (Pmf >47%)	2.85 (0.87–9.38)
Mandro et al, 2015, Salambongo, Wanieruklula, Tshopo Province, DRC [19]	Hyper	13	Hyper	1.89 (0.84–4.28)
Colebunders et al, 2014, Titule, Bas Uélé Province, DRC [20]	Meso	14	Meso	1.30 (0.32–5.30)

^#^OR, unadjusted odds ratio.

Meso, meso-endemic (35% < Pmf <60%); Hyper, hyper-endemic (Pmf >60%).

*144 study participants: Only 1/72 case and 4/72 controls were skin snip negative.

CDTI, community-directed treatment with ivermectin; DRC, Democratic Republic of Congo; NA, not available; Pmf, prevalence of microfiladermia; VC, vector control.

In the Mbam valley of Cameroon, children aged 5 to 10 years from 25 villages had been tested for the presence and density of microfilariae in their skin snips in 1991 to 1993; our team revisited the previously surveyed villages and traced most of these children in 2017 (25 years later) [17]. Individual microfilariae densities during the initial parasitological surveys were strongly associated with the development of epilepsy later in life, with an adjusted incidence ratio of 28.5 (95% CI = 3.84 to 211.27) for developing epilepsy when childhood microfilariae densities exceeded 200 microfilariae/skin snip compared to individuals without detectable microfilaridermia in their skins during their early years [17]. A second cohort study conducted in other parts of Cameroon found similar results, further highlighting the role of *O*. *volvulus* in increasing the risk for epilepsy [18].

All studies showing an association between onchocerciasis and epilepsy need to be carefully examined for possible confounders. However, no consistent confounding factor has been reported that can explain the high prevalence of epilepsy observed in many onchocerciasis endemic areas across Africa. In Uganda, war, the internal displaced person (IDP) camps, and malnutrition could have played a role but these factors cannot be reported in most other onchocerciasis-endemic regions. Neurocysticercosis could be a confounder in the DRC, Tanzania, and Cameroon but not in Maridi (South Sudan) where there are no pigs for cultural reasons. In Maridi, we now have very strong arguments suggesting that the OAE/NS epidemic started after the building of the Maridi dam, a blackly breeding site [37].

Given the high OR observed in case-control studies without previous ivermectin exposure in onchocerciasis meso- and hyper-endemic villages, and the very high incidence ratio observed in the cohort studies, we should consider the first Bradford Hill criterion to be met.

### Consistency

Causation is more likely if the results from various research studies are consistent. This criterion requires examining all studies included in the review to see whether similar conclusions have been drawn. As early as 1938, Casis-Sacre described a syndrome characterized by epileptic seizures, stunted growth, and mental retardation in patients with onchocerciasis in the Chiapas and Oaxaca foci of Mexico [1]. Positive associations between onchocerciasis and epilepsy have been demonstrated in *O*. *volvulus*-endemic areas throughout West [38–40], Central [19,41–43], and East Africa [9,33,44,45]. Although the epileptogenic role of neurocysticercosis was suggested as an explanation in some areas [46], case-control studies did not reveal a significant difference in the prevalence of *Taenia solium* antibodies between cases and controls in those areas [47]. Other authors, analyzing case-control studies in Uganda and South Sudan where NS incidence had increased in the decade preceding their study, concluded that there was a consistent, yet “enigmatic” association with onchocerciasis (detected by skin snip or serological diagnosis) [48].

In Tanzania, the prevalence of epilepsy was 3.5% in 2 rural villages located close to a blackfly-infested river compared to 1.5% in 2 suburban villages (*P* < 0.001) [49] despite 20 years of community-directed treatment with ivermectin (CDTI). The use of a rapid diagnostic test, applied to children aged 6 to 10 years, detected a higher seroprevalence of IgG4 antibodies against the Ov16 antigen of *O*. *volvulus* in these rural villages (42.6%) compared to the suburban villages (4.7%), *P* < 0.001, indicating greater exposure to onchocerciasis in the villages with a higher prevalence of epilepsy [49].

A multisite study of active convulsive epilepsy was conducted in 5 sub-Saharan Africa Health and Demographic Surveillance System (HDSS) sites, including onchocerciasis-endemic sites in 3 countries (Ghana, Tanzania, and Uganda) [50,51]. In 2 of them (Ghana and Tanzania), IgG4 seropositivity to Ov16 antigen by ELISA (sensitivity 90%; specificity 98%) was statistically and positively associated with epilepsy in children (aged <18 years) and adults (18 years and older). Among children <18 years, the population attributable fraction of epilepsy due to *O*. *volvulus* seropositivity was significant in Ghana (Kintampo HDSS = 0.14, 95% CI = 0.01 to 0.25) and Tanzania (Ifakara HDSS = 0.09, 95% CI = 0.01 to 0.17), but not in Uganda (Iganga-Mayuge HDSS = 0.05, 95% CI = 0 to 0.10) [50]. The Iganga-Maguye HDSS in Uganda comprises of districts which were no longer endemic for onchocerciasis, in contrast with Kintampo HDSS in Ghana, and Ifakara HDSS in Tanzania where onchocerciasis was still endemic at the time of the study. However, we must note that in this large study, simultaneous exposure to *O*. *volvulus* and other parasites including *Toxoplasma gondii* increased the risks for convulsive epilepsy [52]. In case-control studies in South Sudan and Uganda, both *O volvulus* and *Mansonella perstans* antibodies were more prevalent in NS cases than in controls [8,9] but this was not observed in a case-control study in an onchocerciasis endemic area in the Bas Uélé Province in the DRC [20]. Moreover, in a recent cohort study in Cameroon, childhood infection with *M*. *perstans* was not found to be a risk factor for developing epilepsy later in life, in contrast to infection with *O*. *volvulus* [18].

A high prevalence and incidence of epilepsy was observed particularly in onchocerciasis-endemic regions where *O*. *volvulus* transmission was either poorly or not at all controlled [3,9,22,41,43,53–55]. In a study of 23 villages in a rural area of Cameroon, the closer a village was to the Mbam river (a known habitat for simuliid species such as those in the *Simulium squamosum* group, which are competent vectors of *O*. *volvulus*), the higher the prevalence of epilepsy (Pearson’s correlation coefficient *r* = 0.465, *P* = 0.026) [3]. In Titule, in the Bas-Uélé Province of the DRC, proximity of households to blackfly-infested rivers was also a risk factor for epilepsy, with increasing distance being negatively associated with the prevalence of epilepsy (OR = 0.63, 95% CI = 0.45 to 0.91, *P* < 0.05) [56]. In the Central African Republic, a study conducted in 1996 reported a positive and significant correlation (*r* = 0.73; *P* < 0.001) between onchocerciasis endemicity levels determined by community microfilarial levels (hypoendemic, mesoendemic, and hyperendemic) and epilepsy prevalence (respectively, 0.5%, 0.8%, and 2.5%), and a negative and significant correlation (*r* = –0.34; *P* < 0.03) between epilepsy prevalence and mean distance from the nearest river of the villages in each endemicity level (respectively, 9.5, 5.9, and 2.4 Km) [57]. A positive correlation between epilepsy and onchocerciasis, albeit being weaker, was also observed in the Imo river basin in Nigeria: (*r* = 0.38; *P* < 0.043) [39,58].

The low number of reports about high epilepsy prevalence in onchocerciasis-endemic regions in West Africa is most likely explained by the success of OCP that was started in 1974 to 1975 [59]. In 1981, a high prevalence of epilepsy in West Africa was reported from an onchocerciasis-endemic region in Liberia (4.9%) [38], a country that was not under the umbrella of the OCP. In contrast, in 1990, in an area of Burkina Faso that was previously hyper-endemic but had become hypo-endemic under OCP [59], the prevalence of onchocerciasis in PWE was not significantly different from that in persons without epilepsy (*P* = 0.67), and the epilepsy prevalence was 1.5% (95% CI = 0.9% to 2.3%), was similar to the 1.1% recorded in a non-endemic area [25]. A recent meta-analysis of epilepsy prevalence studies performed in West Africa showed that before and during the early years of implementing onchocerciasis control in West Africa, high onchocerciasis endemicity was associated with a high prevalence of epilepsy and that subsequent control efforts significantly reduced the prevalence of epilepsy. Higher pre-control endemicity and a shorter duration of onchocerciasis control were both associated with increased epilepsy prevalence (*P* < 0.001) [40]. By applying the OAE criteria on the epidemiological and clinical data obtained from 2 epilepsy studies in Ivory Coast [60,61], we found that >70% of PWE in the study villages reported features of OAE. It is therefore plausible that these sites were OAE hotspots at the time of the study, when onchocerciasis transmission was still high [40].

The notion that OAE may have been a possible cause of excess mortality in the West African countries covered by the OCP is suggested by the study of Walker and colleagues [62] in which 295,909 persons with onchocerciasis recorded in the OCP cohort database were follow-up for 25 years: The relative risk of mortality increased with increasing skin microfilariae load and this risk was significantly higher in those aged <20 years compared to those ≥20 years old [62]. Onchocercal eye disease cannot explain these excess deaths in younger people, as it is more frequent in adulthood [63]. Given that seizure onset between the ages of 3 to 18 years is characteristic for both NS [48] and OAE [64], we surmise that the high mortality observed among those <20 years old could be due to premature mortality in PWE living in these areas [65,66].

While a statistically significant association between onchocerciasis and epilepsy has been demonstrated in areas where onchocerciasis is meso- or hyper-endemic [3,5,9,54], some case-control studies in settings with lower endemicity have not reported such an association [25,34,67]. This could be due to the fact that epilepsy is a chronic condition which persists even after treating or controlling onchocerciasis. Moreover, epilepsy onset is often accompanied by dramatic life changes such as decreased exposure to the river and blackflies (because of the risk of drowning in the river) and increased ivermectin use as was observed in the DRC [41,68]. Consequently, these factors may influence results regarding *O*. *volvulus* microfilaridermia status and microfilarial density in the case-control studies which enroll PWE with a certain duration of epilepsy [56].

In the case-control study in the Bas-Congo Province in the DRC, an association between onchocerciasis and epilepsy was found (Table 2), but when stunting was included as a correcting covariate, the relationship between onchocerciasis and epilepsy was no longer significant [32]. The results of this study are, however, difficult to interpret; indeed only 22 controls were included, there was no information about previous ivermectin use, and there is a problem of multicollinearity between epilepsy and stunting as both conditions have been reported to be associated with onchocerciasis [64].

In summary, the association between epilepsy and onchocerciasis has been observed in a variety of settings and populations, spanning from conflict zones with displaced populations and episodes of food insecurity to stable settings with adequate food supply. Study results not showing an association between onchocerciasis and epilepsy/NS can be explained by low onchocerciasis endemicity, prior onchocerciasis control interventions including ivermectin treatment, not taking into account participants’ microfilarial load during the data analysis, and/or recruiting controls with onchocerciasis-associated morbidity. As 100% of studies not meeting the latter descriptions support the association, this criterion is met.

### Specificity

**Fig 2:**
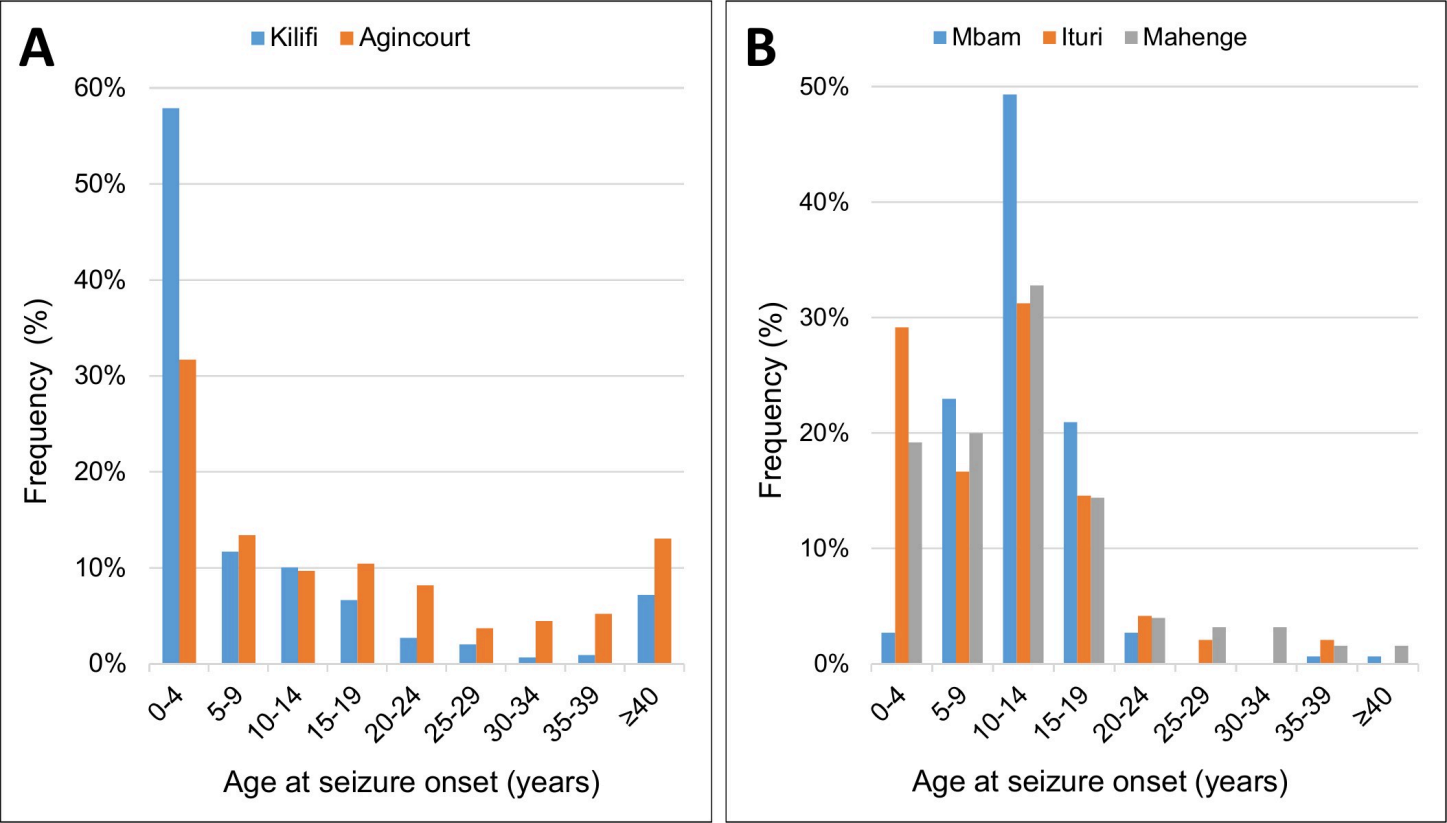
The ages of village residents at epilepsy onset (A) in non-onchocerciasis–endemic regions in Kilifi, Kenya and Agincourt, South Africa [50] (B) in onchocerciasis-endemic communities in Mbam, Cameroon [43], Ituri, DRC [53], and Mahenge, Tanzania [49].

According to Bradford Hill, causation is more likely if there is a specific outcome related to a specific exposure. Onchocerciasis indeed seems to be associated with a specific type of epilepsy with an onset of seizures of unknown etiology in previously healthy children between the ages of 3 to 18 years (with a peak onset between the ages 8 to 12 years), with some children presenting with nodding seizures and Nakalanga features [44,64,69–71]. This age of epilepsy onset between 3 and 18 years, although not exclusive to OAE, is very characteristic and is not frequently observed in non-onchocerciasis–endemic regions in Africa [50]. In the latter, most people develop epilepsy before the age of 5 years due to perinatal causes or epilepsy of genetic origin [50] (Fig 2).

**Fig 3:**
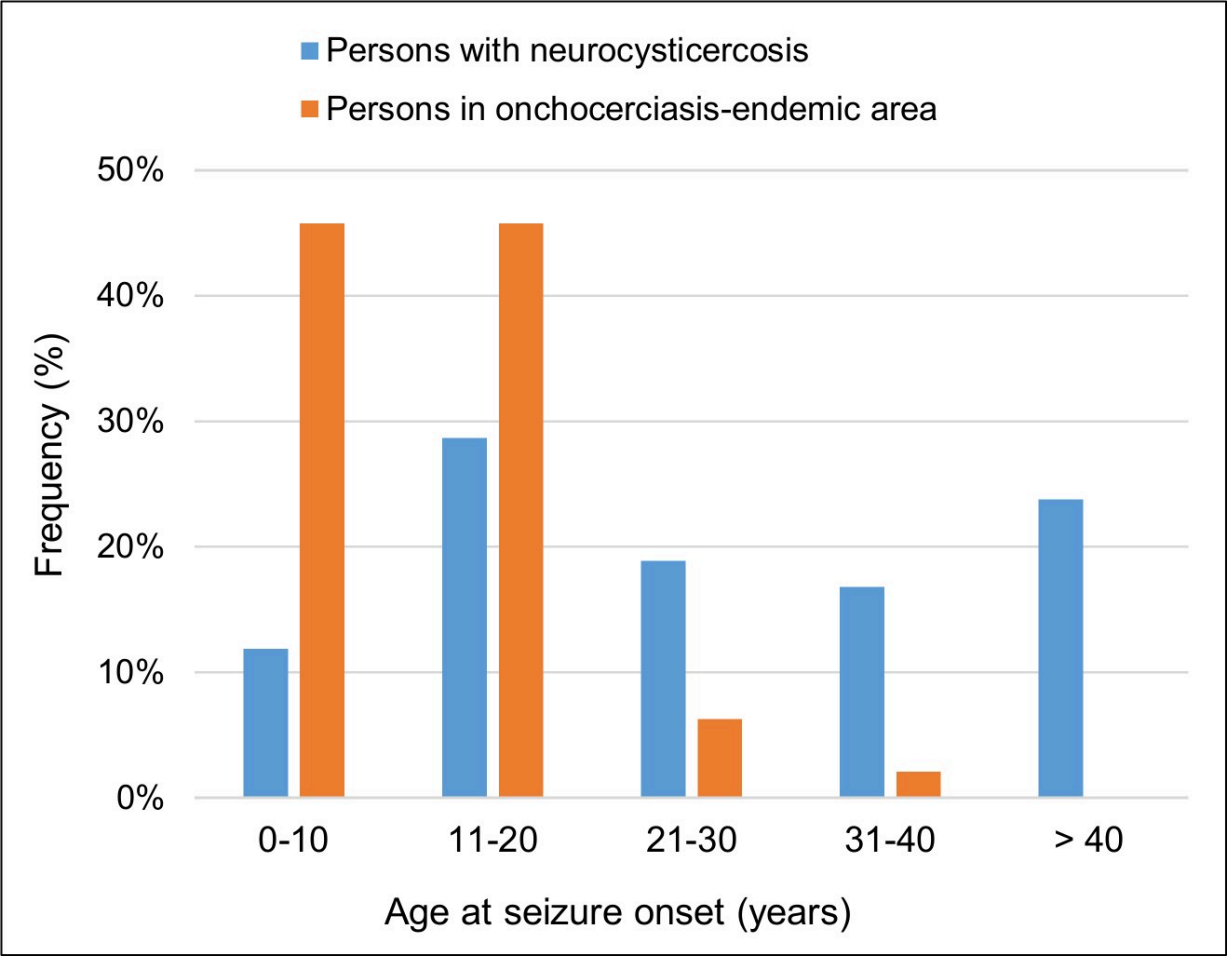
The ages at seizure onset in persons with neurocysticercosis (blue) and those in onchocerciasis-endemic areas (orange). Adapted from findings reported by Monteiro and colleagues [72] and by Lenaerts and colleagues [53].

The pattern of age at OAE onset is also different from that of seizures caused by neurocysticercosis. Neurocysticercosis is characterized by a later onset of epilepsy with more persons experiencing their first seizure after the age of 20 years [72] (Fig 3).

The data on epilepsy prevalence in onchocerciasis-endemic regions need to be interpreted with caution in the absence of additional tests such as neuroimaging to differentiate OAE from epilepsy due to neurocysticercosis and other causes. However, in a recent epilepsy survey of 17,652 persons living in Maridi county, an onchocerciasis-hyper-endemic area in South Sudan where there are no pigs, an epilepsy prevalence of 4.4% was observed with more than 85% of PWE meeting the criteria of OAE (≥2 seizures without any obvious cause, starting between the ages of 3 to 18 years in previously healthy persons who had resided for at least 3 years in an onchocerciasis meso- or hyper-endemic area) [54]. This high percentage of epilepsy in Maridi, most likely caused by OAE, is similar to the estimated high population attributable fraction of OAE (91.7%, 95% CI 56.7 to 98.4; *P* = 0.0021) in the cohort study in Cameroon [17]. Additionally, a second cohort study ruled out infection with *Loa loa* and *M*. *perstans* as the cause of the epilepsy burden observed in Cameroonian villages [18].

Causation is also more likely if a specific population, at a specific site, presents with the condition and when no other likely explanation exists beyond the suspected trigger. In other words, the specificity criterion relates to whether the type of epilepsy in onchocerciasis-endemic regions could be attributed to anything other than onchocerciasis. NS and Nakalanga syndrome have only been reported in onchocerciasis-endemic areas, particularly in villages where onchocerciasis is hyper-endemic and together with a high prevalence of other forms of epilepsy [64]. A suspected case of NS was reportedly seen in India [73], but the clinical presentation was substantially marred by preexisting neurological conditions, thus making the diagnosis of NS very unlikely. While other infectious, nutritional, or environmental causes have been suggested for the NS [74,75], the most significant association was consistently found with *O*. *volvulus* infection [76].

**Fig 4:**
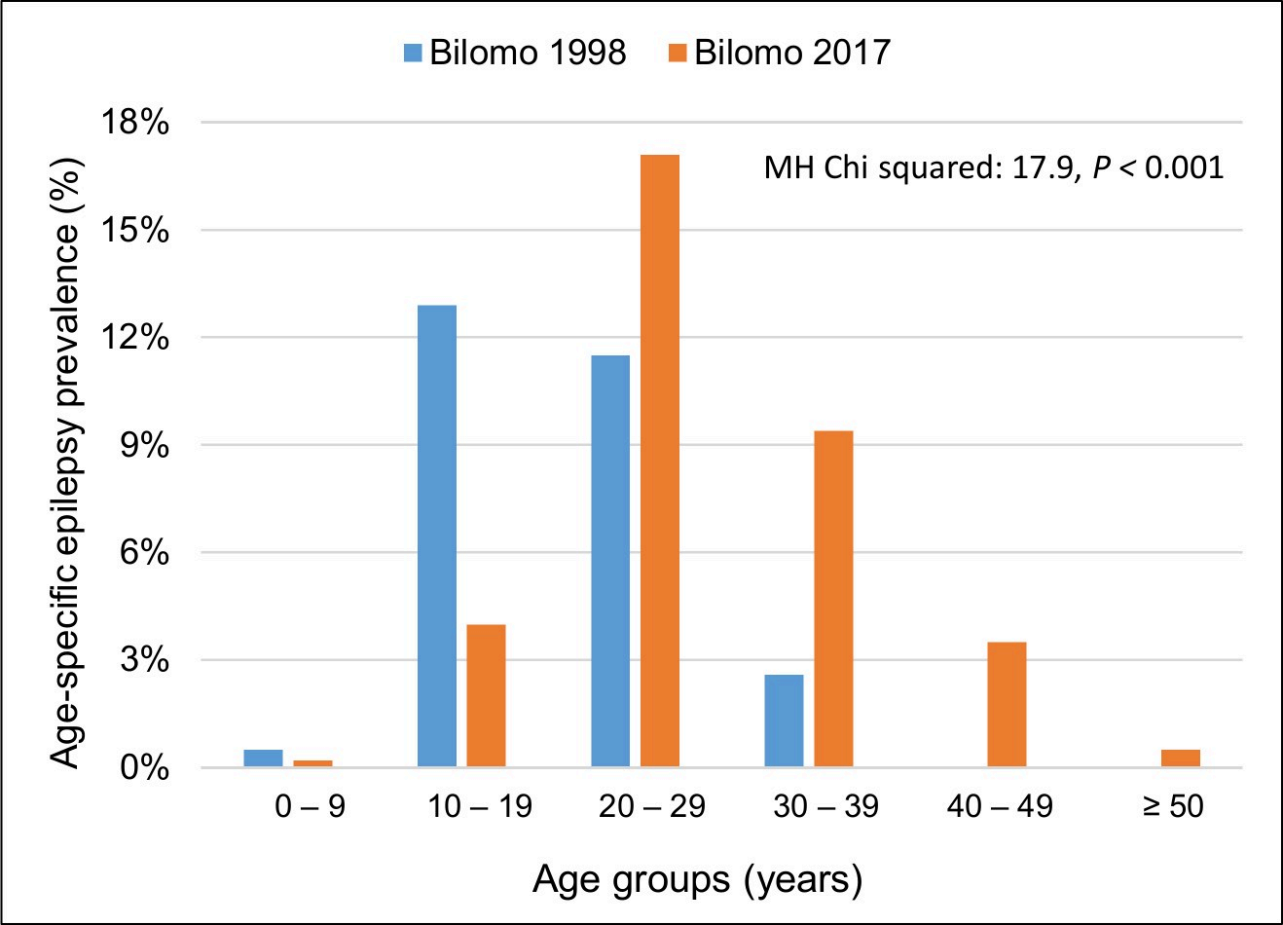
Comparison of age-specific crude prevalence of epilepsy in Bilomo, Mbam valley, Cameroon, 1998 (blue) versus 2017 (orange) [43]. MH, Mantel–Haenszel.

According to Bradford Hill, causation is more likely if altering the cause modifies the disease outcome. This criterion also seems to be met as onchocerciasis elimination efforts have been shown to decrease the incidence of epilepsy [77], including NS [43,78]. Indeed, in northern Uganda (Kitgum, Pader, and Lamwo districts), no new cases of NS have been reported after the implementation of biannual treatment with ivermectin coupled with ground-based larviciding of the rivers in which the vectors breed since 2013 [78]. This is in contrast with South Sudan, where CDTI has frequently been interrupted and the NS epidemic is ongoing [37,54]. In several villages in the Mbam valley in Cameroon, an age shift toward older PWE has been observed; in fact, the peak age-specific prevalence of epilepsy shifted from the 5 to 19 years age group to 20 to 39 years age group after 19 years of CDTI [42,43] (Fig 4).

This shift to older ages suggests a decreased incidence of OAE in the 5 to 19 years age group. Improvement of perinatal and child care in Bilomo cannot explain this increase in age because since 1998, the prevalence of epilepsy in the children aged below 10 years was already very low. A similar age shift was observed in 2017 in northern Uganda [78]. These findings, however, should be confirmed in a prospective, population-based study.

In 1994, a study in Kabarole, an onchocerciasis-endemic focus in western Uganda documented a prevalence of epilepsy of 3.0%, and reported cases of NS [79]. Following the elimination of onchocerciasis in this area since 2004, the same villages were revisited in 2018 and no new cases of NS were found among persons who were never exposed to *O*. *volvulus*. We equally observed a significant drop in the overall epilepsy prevalence (from 3.0% to 1.2%) and incidence after onchocerciasis elimination (from 418 to 73 new cases per 100,000 person-years), with the greatest impact on the 10 to 19 years age group which is usually the most affected by OAE [79]. A thorough overview of possible changes in the common risk factors for epilepsy in the Kabarole villages (perinatal brain insult, cerebral malaria, neurocysticercosis, and genetics) could not explain the substantial reduction in the epilepsy burden, leaving the elimination of onchocerciasis as the most plausible explanation [79].

It is noteworthy that only a minority of children with *O*. *volvulus* infection develop epilepsy; more often they develop skin manifestations such as itching and dermatitis [80]. Long-term exposure to high microfilarial loads is necessary to become blind, with the incidence of blindness being significantly and positively associated with increasing microfilarial load [81]. The threshold microfilarial density for developing epilepsy is probably lower than that for blindness (>20 microfilariae/skin snip has been suggested, above which there is severe ocular morbidity [82]), although it likely still needs to be sufficiently high [17,83]. Hence, the different clinical presentations of OAE (NS, Nakalanga syndrome, and other forms of epilepsy) may be related to the age at which children become infected with *O*. *volvulus* and their microfilarial density. In a recent study in South Sudan, persons with NS were found to have more disabilities and to have higher microfilarial densities than persons with other forms of OAE [84]. In areas where *Onchocerca ochengi* is endemic among cattle, humans when bitten by *O*. *ochengi*-infected blackflies may develop some immunity toward *O*. *volvulus* [85,86], and therefore, in such areas, children may develop lower *O*. *volvulus* microfilarial loads and consequently less NS and Nakalanga which are characterized by high microfilarial densities. The fact that other zoonotic filarial infections may play a similar protective role is not excluded and may influence the clinical spectrum of OAE. Genetic diversity of the *O*. *volvulus*, human genetics, immunological, and nutritional risk factors as well as other coinfections could also be incriminated. Although the available evidence supports that *O*. *volvulus* infection may cause epilepsy, more studies are warranted to better investigate in detail other confounding epilepsy etiologies in onchocerciasis foci. We do not consider this criterion to be met.

### Temporality

Until recently, all studies investigating the association between onchocerciasis and epilepsy were cross-sectional and therefore unable to demonstrate the temporal nature of causality. A first retrospective cohort study conducted in the Mbam valley of Cameroon showed that children in onchocerciasis-endemic areas first acquire *O*. *volvulus* infection and later develop epilepsy, particularly those with a high microfilarial load, in a dose-response relationship [17]. A second retrospective cohort study was performed in the Lékié division, an area of Cameroon where *L*. *loa* and onchocerciasis are co-endemic, which confirmed the temporal relationship between onchocerciasis and epilepsy [18].

At the population level, an NS epidemic started around the year 2000 in the districts of Kitgum, Pader, and Lamwo in northern Uganda, in an onchocerciasis-endemic region where ivermectin was not distributed. By 2008 after initiation of CDTI, the number of new NS cases started to decrease [78]. The reason why an NS epidemic started in northern Uganda is most likely multifactorial. Contributing factors to consider are the close proximity of IDP camps to the blackfly breeding sites, no access to ivermectin, and the poor nutritional status of the children during the war, making them susceptible to more severe *O*. *volvulus* infection as well as other health conditions. Another event that may have played a role is the theft in 1986 of about 300,000 cattle from the Acholi people in northern Uganda [87]. The presence of a large number of cattle in a village may decrease *O*. *volvulus* transmission and onchocerciasis-associated morbidity. Indeed before 1986, cattle were grazing the high grass along the rivers exposing blackflies to the sun and reducing their lifespan. Moreover, humans will be less bitten by blackflies because blackflies are an alternative food source. Finally, the hypothesized cross-immunity mechanisms that occur when humans are bitten by *O*. *ochengi*-infected blackflies may confer some protection against *O*. *volvulus* [85,86].

In 1948, DJ Lewis, a medical entomologist, described Mvolo in Western Equatoria in South Sudan as a place with extremely intense *Simulium* spp. biting and high infection prevalence in the flies (up to 10% of flies with L3 larvae in the heads) [88]. He described Mvolo as having only a police post with very few people residing in the area. Today Mvolo is a rural city, where a rapid assessment revealed that about 50% of the families have at least 1 child with epilepsy [89]. It is hypothesized that despite the risk for onchocerciasis, many people settled in Mvolo because of the fertile grounds located close to the Naam river and because of the great fishing opportunities. In a recent door-to-door survey in Maridi county in South Sudan, 692 (4.7%) of 4,619 permanent household members were found to have epilepsy compared to 82 (2.7%) of 3,033 immigrant household members; among the latter group, the prevalence of epilepsy increased with increasing duration of residence in the endemic village [54]. We consider this Bradford Hill criterion to be met.

### Biological gradient (dose response)

The severity of the cause should be positively correlated with the severity of the effect. This criterion appears to be met. Indeed, the risk for an individual of developing epilepsy increases in a dose-related manner with increasing microfilarial load [17,18] (as does the relative risk of mortality in children aged <20 years [62]) in onchocerciasis-endemic (or previously endemic) areas. At the community level, a higher prevalence of epilepsy has been observed in villages in Cameroon with a higher mean community microfilarial load [3]. Based on a meta-analysis of 8 population-based epilepsy surveys performed in 7 different countries, Pion and colleagues [5] calculated that, on average, the prevalence of epilepsy increased by 0.4% for each 10% increase in onchocerciasis prevalence. A higher microfilarial density was found to be associated with NS, a more severe form of OAE [84].

Onchocerciasis elimination efforts, which lead to reductions in microfilarial prevalence and load, have been shown to decrease the incidence of epilepsy/NS [78,79]. Recent studies in the DRC showed that high seizure frequency was associated with increasing microfilarial density [71] and increasing levels of urinary N-acetyltyramine-O,β-glucuronide (NATOG), a biomarker for onchocerciasis [90]. Furthermore, a randomized trial in the DRC, comparing a single versus a multiple dose of ivermectin treatment regimen in *O*. *volvulus*-infected persons with epilepsy treated with phenobarbital showed that the multiple dose ivermectin regimen was associated with a reduced frequency of seizures [91]. The PWE included in this trial were all initially treated with phenobarbital despite having different seizure types, which may have affected the overall study outcomes. Given the complexity of the trial, these results need to be interpreted with caution. However, this trial certainly shows that treatment with ivermectin is not a possible confounder that could explain the high prevalence of epilepsy in onchocerciasis-endemic regions. We consider this Bradford Hill criterion is met.

### Plausibility

This criterion is met if a hypothesized effect makes sense in the context of current biological knowledge. There are 2 plausible biological mechanisms that could explain how *O*. *volvulus* infection is able to cause epilepsy/NS. The first mechanism is that *O*. *volvulus* infection induces the production of neurotoxic autoantibodies. Recently, it was shown that leiomodin-1 antibodies were more often present, and at higher levels, in sera of Ugandan and South Sudanese children with NS compared to controls [92]. These antibodies were also present in the cerebrospinal fluid of children with NS. They were found to be neurotoxic in vitro and cross-react with *O*. *volvulus*-specific proteins (a phenomenon known as molecular mimicry) [92]. The leiomodin-1 protein is expressed in specific neuronal populations in the brain, including cortical neurons in the CA3 region of the hippocampus, and the Purkinje cells in the cerebellum [93]. However, leiomodin-1 is a member of the actin filament nucleator family that is highly enriched in smooth muscle-containing tissues such as the artery wall and the gastrointestinal tract [94]. If NS was an autoimmune disease triggered by leiomodin-1 antibodies, one would expect symptoms related to vasculitis, which is not reported in NS and was not observed in 2 postmortem studies [95,96]. So far, the Leiomodin-1 hypothesis has not been confirmed and therefore cannot be used as an argument for plausibility.

A recently reported case-control study including 30 South Sudanese persons with NS and a similar number of healthy participants from the same geographical region revealed autoimmune antibodies to 3 extracellular peptides of ionotropic glutamate receptors in NS patients: AMPA-GluR3B peptide antibodies (86%), NMDA-NR1 peptide antibodies (77%), and NMDA-NR2 peptide antibodies (87%) [97]. However, these antibodies were also observed in some controls at a lower concentration and were previously found in many more patients with other types of epilepsy and neurological conditions [98]. Therefore, the connection between these antibodies and NS is not clear. The same research group showed that in South Sudan, NS was associated with both protective HLA haplotypes: HLA-B*42:01, C*17:01, DRB1*03:02, DQB1*04:02, and DQA1*04:01, and the susceptible motif: Ala24, Glu63, and Phe67, in the HLA-B peptide-binding groove. The authors therefore suggested that different HLA molecules may explain why under similar environmental conditions, only some children within the same families, tribes, and districts would develop NS, while others do not [99].

A second mechanism that needs to be explored is that *O*. *volvulus* microfilariae themselves or parasite-derived factors can cross the blood–brain barrier and cause neuronal damage, either due to their direct neurotoxicity or by provoking a secondary inflammatory response. Before the introduction of CDTI, the presence of *O*. *volvulus* microfilariae in the cerebrospinal fluid was reported in heavily infected patients, particularly after treatment with diethylcarbamazine [6,7,100]. However, recent studies on cerebrospinal fluid of patients with NS and epilepsy in onchocerciasis-endemic regions have failed to identify *O*. *volvulus* microfilariae or DNA [35,76,101,102].

A recently published postmortem study of 5 persons from northern Uganda who died of NS between 2014 and 2017 suggested that NS is a tauopathy and a neurodegenerative disease [95]. However, in another postmortem study performed in 2017 to 2018, among 5 persons with NS and 4 persons with another form of OAE who died in the same region, no evidence of a tauopathy was revealed [96]. Therefore, tau deposits are most likely the consequence and not the cause of the disease. In fact, tau pathology can be induced by seizures themselves [103] as well as by seizure-associated phenomena including hypoxia [104] and repeated head injuries [105]. We do not consider that this Bradford Hill criterion is met.

### Coherence

Causation is more likely if clinical observations are supported by and in agreement with the natural history of the disease. After becoming infected with *O*. *volvulus* and without ivermectin treatment, microfilarial loads will increase over time because exposure to infection and reinfection continues [63], and the adult parasite is very long-lived. It is mainly between the ages of 3 to 18 years, during the period in which children develop OAE, that the rate of increase in microfilarial load is more pronounced [81]. We surmise that around a median age of 8 years, some children, already heavily infected at a young age, may develop NS associated with severe cognitive impairment [48,106], while others, less heavily infected, and/or infected at a later age, may develop other forms of epilepsy associated with less disability around the age of 11 years [106]. Similar to other infectious diseases, not all persons develop the disease after even a major exposure to a certain pathogen. It is possible that individuals who do not develop epilepsy once a certain threshold of microfilarial density is reached, for genetic and/or immunological reasons, will never develop epilepsy later.

Furthermore, between 3 and 18 years, a large neuronal remodeling occurs in the brain of children and adolescents, with the formation of new neuronal connections and disappearance of others. It is possible that during this period, brain cells are particularly vulnerable.

We do not consider that this Bradford Hill criterion is met.

### Experimental evidence

*O*. *volvulus* infections only occur in humans, and this is a challenge for the development of a suitable animal model. Regarding evidence potentially provided by clinical trials, it is not ethically possible to conduct a prospective cohort study comparing the incidence of epilepsy in *O*. *volvulus-*infected individuals treated or not treated with ivermectin. However, a prospective study is planned in South Sudan to demonstrate whether implementation of ivermectin twice yearly (biannual CDTI) will decrease the incidence of OAE in a highly endemic area [107].

We do not consider that this Bradford Hill criterion is met.

### Analogy

As stated above, *O*. *volvulus* infection only causes disease in humans. *O*. *ochengi*, a parasite of cattle and the closest relative of *O*. *volvulus* [108], is transmitted by the same blackfly vector species (*Simulium damnosum sensu lato*) but is not known to cause epilepsy. Other species of *Onchocerca* can cause disease in animals and sometimes in humans (e.g., *O*. *lupi*), but thus far, these infections have not been associated with epilepsy [109]. Only one of the other filarial infections (*L*. *loa*) is able to cause encephalopathy, but this is related to microfilariae dying in the peripheral circulation mostly as a result of microfilaricidal treatment of heavily infected individuals. A similar phenomenon is unlikely in onchocerciasis because, in contrast with loasis, *O*. *volvulus* microfilariae are not usually present in blood [80,110].

Hookworm infections, like onchocerciasis, are acquired during the childhood, when the exposure is high. They both seem to affect the childhood development and some cognitive functions [111]. Also, for pinworms (*Enterobius vermicularis*), there is an old known effect on the behavior of heavily infected children [112].

*O*. *volvulus* may cause epilepsy in a similar way as it causes blindness. Lesions of the anterior part of the eye are directly caused by the dying microfilariae, and probably also by an inflammatory reaction toward the endosymbiont *Wolbachia* released by dying microfilariae, as both the filariae and endobacteria contribute to the pathogenesis of onchocerciasis [113]. The pathophysiological mechanism that causes the retinal lesions observed in *O*. *volvulus*-infected persons remains, however, poorly understood. The incidence of these ocular lesions increases and the average age of onset decreases as the intensity of transmission and infection in the community rises [114]. Microfilariae have been observed in the retina [115] but it has also been suggested that retinal lesions are caused by an autoimmune process whereby *O*. *volvulus* antibodies react with retinal proteins [116]. The *O*. *volvulus* antigen Ov39 is cross-reactive with the retinal antigen hr44 and induces ocular inflammation in rats after immunization [117]. A similar mechanism of molecular mimicry may play a role in NS as discussed with leiomodin-1 and *O*. *volvulus* proteins [92]. In retinal onchocerciasis, the inflammatory process can continue and visual impairment persists despite ivermectin treatment. In OAE, it still needs to be determined whether early antiepileptic and antifilarial treatment may alter or even reverse the observed progressive evolution toward an encephalopathy in some individuals. We do not consider that this Bradford Hill criterion is met.

## Discussion

This paper reviews current evidence for the causal association between epilepsy/NS and onchocerciasis. Onchocerciasis as a risk factor for epilepsy meets the following Bradford Hill criteria for causality: strength of the association, consistency, temporality, and biological gradient. There is also weaker evidence for causality concerning the criteria: specificity, plausibility, coherence, and analogy. Until now, there is little experimental evidence, and this is identified as an important research gap that needs further in vivo, in vitro, and epidemiological studies such as those discussed here [92,107,118,119]. However, consideration of the Bradford Hill criteria suggests that there is consistent evidence that *O*. *volvulus*, in the presence of a sufficiently high microfilarial load, is able to trigger epilepsy including NS and Nakalanga features. Onchocerciasis is associated with a large number of clinical manifestations that include not only skin and ocular lesions, but also different types of epilepsy, intellectual disabilities, stunted growth, facial, thoracic, and spinal abnormalities, and delayed or absence of sexual development. *O*. *volvulus*-infected individuals may present with one or a combination of these manifestations [64].

Our extensive literature review pointed out the confounding role of CDTI during previous studies investigating this subject, because frequent ivermectin use has the ability to mask an association between onchocerciasis and epilepsy [19]. While debated in the epidemiological community [120], the Bradford Hill criteria are still widely accepted as useful guidelines for investigating causality in epidemiological studies [121]. As the world of epidemiological research is evolving, our criteria for determining causal inference must similarly evolve to reflect the multidisciplinary research needed to establish a causal association. Further research is needed to identify cofactors, such as parasitic coinfections, nutritional and (epi)genetic factors that may increase the risk for *O*. *volvulus* infected-children to develop epilepsy. The OAE definition mentioned severally in this paper is a useful tool to estimate the burden of disease caused by onchocerciasis besides skin and eye disease. Although of little diagnostic value, OAE criteria offer a convenient public health approach to identifying hotspots where onchocerciasis elimination efforts need to be strengthened [122]. However, to identify the pathophysiological mechanism of OAE, the preferred study population should be persons with NS since it is a specific, typical, and well-characterized onchocerciasis-related epileptic syndrome [123]. Based on studies with other nematode infections, we can hypothesize that certain *O*. *volvulus* excretory/secretory molecules may have an effect on the human brain and its functions. Different cofactors may be involved in different onchocerciasis-endemic regions, explaining differences in the epidemiology and clinical spectrum of OAE. Screening for neurotoxic autoantibodies or *O*. *volvulus*-secreted proteins in blood and cerebrospinal fluid samples of persons with new onset epilepsy in onchocerciasis-endemic regions and controls, as well as additional postmortem studies of persons who died during an earlier phase of the disease, may be the way to identify the pathophysiological mechanism(s) by which *O*. *volvulus* may cause epilepsy/NS. Ultimately, we may need an experimental animal model to confirm this (these) mechanism(s).

### Conclusions

Applying the Bradford Hill criteria, there appears to be consistent epidemiological evidence that *O*. *volvulus* infection can cause epilepsy, including NS and Nakalanga features. The pathophysiological mechanism by which *O*. *volvulus* affects the central nervous system is unclear and represents a research gap in this domain. Although understanding the underlying biological mechanism of OAE is important, it should not stop us from taking appropriate public health action [124]. Bradford Hill himself, stated “what is biologically plausible depends upon the biological knowledge of the day” [11]. Therefore, while this enigma is being solved and onchocerciasis eventually becomes known as “river epilepsy” rather than “river blindness,” public health efforts must be stepped up to effectively control and eliminate onchocerciasis and to manage epilepsy in the affected and suffering populations.

## Supporting information

S1 Table Search strategy for papers reporting epilepsy, nodding/Nakalanga syndrome, and onchocerciasis in PubMed (search date: September 15, 2020). (PDF)

S2 Table Literature retrieved via PubMed and manual searchesTab. (XLSX)
