# Supplementary material for: Onchocerca volvulus and epilepsy: A comprehensive review using the Bradford Hill criteria for causation
Source: PLoS Negl Trop Dis. 2021 Jan 7;15(1):e0008965. doi: 10.1371/journal.pntd.0008965 (PMC7790236; doi:10.1371/journal.pntd.0008965)
Supplement: S1 Table — (PDF) [file pntd.0008965.s001.pdf]

Search strategy for papers reporting epilepsy, nodding/Nakalanga syndrome, and onchocerciasis in PubMed

(Search date: 15th September 2020)

No.	Search terms	Search details on PubMed	Hits
#1	Epilepsy (Mesh term)	"Epilepsy"[Mesh]	111,777
#2	Epilepsy (free text)	epilep*[TIAB]	141,206
**#3**	**Final search: epilepsy**	("Epilepsy"[Mesh]) OR epilep*[TIAB]	165,455
#4	Nodding syndrome (Mesh term)	"Nodding Syndrome"[Mesh]	76
#5	Nodding and Nakalanga syndromes (free text)	(Nodding[TIAB] OR Nakalanga[TIAB]) AND (syndrome*[TIAB] OR seizure*[TIAB] OR feature*[TIAB])	227
**#6**	**Final search: Nodding and Nakalanga syndromes**	("Nodding Syndrome"[Mesh]) OR ((Nodding[TIAB] OR Nakalanga[TIAB]) AND (syndrome*[TIAB] OR seizure*[TIAB] OR feature*[TIAB]))	229
#7	Onchocerciasis (Mesh term)	"onchocerciasis"[Mesh]	4,096
#8	Onchocerciasis (free text)	onchocerc*[TIAB]	5,439
**#9**	**Final search: onchocerciasis**	("onchocerciasis"[Mesh]) OR onchocerc*[TIAB]	5,897
**#10**	**FINAL SEARCH:**<br>**(#3 OR #6) AND #9**	((((("Epilepsy"[Mesh]) OR epilep*[TIAB])) OR (("Nodding Syndrome"[Mesh]) OR ((Nodding[TIAB] OR Nakalanga[TIAB]) AND (syndrome*[TIAB] OR seizure*[TIAB] OR feature*[TIAB]))))) AND (("onchocerciasis"[Mesh]) OR onchocerc*[TIAB])	142
**Total number of articles retained after screening the abstracts**			**66**
